# The effects of reserpine on depression: A systematic review

**DOI:** 10.1177/02698811221115762

**Published:** 2022-08-24

**Authors:** Rebecca Strawbridge, Rahila R Javed, Jeremy Cave, Sameer Jauhar, Allan H Young

**Affiliations:** 1 Department of Psychological Medicine, Institute of Psychiatry, Psychology & Neuroscience, King’s College London, London, UK; 2 South London & Maudsley NHS Foundation Trust, London, UK

**Keywords:** Depression, monoamine, *Rauwolfia serpentina*, reserpine, systematic review

## Abstract

**Background:**

Reserpine is an effective antihypertensive drug, but its role in routine practice has declined such that it is rarely used. This is largely based on the assumption that reserpine causes depression. This assumption was a foundation for the original monoamine hypothesis of depression. However, there remains conflicting evidence as to whether reserpine causes depression, and no systematic review of available evidence.

**Aims:**

We systematically reviewed evidence on effects of reserpine on depressive and related symptoms (e.g. anxiety, suicidal ideation).

**Method:**

Electronic searches of MEDLINE, Embase and PsycINFO were conducted to identify studies up to 14 February 2021. Studies of any methodological design involving reserpine-treated and reserpine-untreated conditions, in any adult human population, were included and a narrative synthesis of findings was undertaken. Risk of bias (RoB) was examined using ROBINS-I.

**Results:**

Of the 35 studies meeting inclusion criteria, 9 were randomised controlled trials. Eleven studies reported some depressogenic effects, 13 reported no effect and 11 reported putative antidepressant effects. Studies identifying depressive effects were more likely to examine people without psychiatric disorders at baseline, while studies identifying a potential antidepressant effect tended to treat fewer participants for shorter durations, at higher doses. Around one-third of studies conducted in people with psychiatric disorders showed beneficial effects on depression symptoms. 30/35 studies were at high RoB.

**Conclusions:**

Associations between reserpine and depression are inconsistent and limited by a lack of high-quality evidence. Due to reserpine’s apparently complex effects, we urge nuance rather than simplicity surrounding the monoamine hypothesis of depression.

## Introduction

Reserpine is an alkaloid extracted from the root of the *Rauwolfia serpentina* plant, which became commercially available in Western medicine in 1952 after being used for centuries in Indian medicine for a variety of illnesses, including schizophrenia (López-Muñoz et al., 2004). Reserpine was used as a first-line antihypertensive with clear efficacy (Shamon and Perez, 2016), including for individuals with refractory hypertension (Siddiqui et al., 2020). Currently, reserpine is considered a second-line treatment (Furberg et al., 2002) but its use fell dramatically following reports of depression after treatment (Healy and Savage, 1998; López-Muñoz et al., 2004).

The first reports of depression in humans, as a potential consequence of reserpine, emerged in the early 1950s. Freis (1954) observed psychiatric complications including sadness, fatigue and suicidal ideation in five patients with hypertension treated with large doses of reserpine. These were not observed in patients taking other antihypertensives, and symptoms ceased after reserpine discontinuation. Other case series supported this, including in patients with a history of psychiatric illness (Nick, 1955). Notably, at this time reserpine was also being used as an antipsychotic treatment and appeared effective in some individuals with refractory schizophrenia (Preskorn, 2007), although it was not universally supported for this indication (Shepherd and Watt, 1956) and its popularity was short lived (Isharwal and Gupta, 2006).

This observation was also one of the foundations for the monoamine hypothesis of depression, suggesting deficiency of monoamines to be linked to depression. As reserpine depletes catecholamines so markedly (see below), and monoamine oxidase inhibitors were found to be beneficial at ameliorating depressive-like symptoms, improvements in depression were thought to be linked to catecholamine increases (Hull and Horita, 1964). Partly as a result of the clinical observations of depression after reserpine, the monoamine hypothesis of depression has persisted in influencing conceptual thinking in behavioural pharmacology (Carlsson, 2001) through to the current day (Blasco-Serra et al., 2015; El-Marasy et al., 2021).

Here, we briefly summarise the mechanism by which reserpine is thought to provoke depression, essentially through catecholamine depletion. Reserpine binds irreversibly to catecholamine storage vesicles, such as dopamine and norepinephrine, and blocks adrenergic neurotransmission by irreversibly inhibiting the vesicular monoamine transporter-2 (VMAT-2). This interference in the adrenergic neurotransmission pathway depletes catecholamine pumps, ultimately leading to inhibited uptake of neurotransmitters into pre-synaptic storage vesicles. This degradation of catecholamines from peripheral and central synapses occurs through intraneuronal monoamine oxidase in the cytoplasm (Cheung and Parmar, 2022).

Nevertheless, there are reasons to question reserpine’s purported depressogenic effects. One argument is that the claims of reserpine-induced depression originated from observations made by physicians other than psychiatrists, and that when assessed by experienced psychiatrists, patients may rarely meet diagnostic criteria for depression (Healy and Savage, 1998). An example is that akathisia is a side effect of reserpine (similar to other neuroleptics) and is notoriously challenging to diagnose, often misconstrued as affective episodes (Akagi and Kumar, 2002; Healy and Savage, 1998); although a lack of association with depression has previously been reported (Halstead et al., 1994). A second argument – more related to a lack of high-quality research – is that pharmaceutical companies may have a conflict of interest in favour of declining reserpine use; the molecular structure of reserpine does not allow for chemical manipulations that can generate further patentable derivatives, so there is incentive for pharmaceutical companies to prioritise other more marketable compounds (Healy and Savage, 1998). Third, it is argued that the ‘depressive syndromes’ observed under reserpine respond to stimulant treatment, indicating a physiological rather than psychopathological effect (Healy and Savage, 1998). Finally, it is argued that psychiatrists’ views should be considered more closely as they have previously heralded its putative benefits both for affective and psychotic syndromes. These arguments do not deny tolerability or safety considerations, rather that reserpine may be like other antipsychotics that can cause tranquilisation and Parkinsonism symptoms when used for longer durations and at higher doses (an approximately 10-fold excess of recommended dose was used in some of the early case series) and can cause akathisia or dyskinesia during early treatment (Healy and Savage, 1998).

This literature has been (non-systematically) reviewed previously: Considering 61 case reports (from 14 studies), a depression rate after reserpine was found from original articles to be 66%, which decreased to an approximately 10% when restricted to the reviewed *group* studies. The authors concluded that many of the depression cases were not necessarily caused by reserpine and that one reason why this has not been established in the literature is a reticence to disregard the monoamine hypothesis of depression (Baumeister et al., 2003).

### Objectives

Given long-standing and substantial clinical consequences which have arisen from the reserpine–depression link, it is surprising that a systematic review of this literature has not been published.

We therefore aimed to systematically review the literature to evaluate all available evidence related to the depression-related effects of reserpine. The primary aim of this review was to indicate the nature and extent of depressive episodes as consequences of reserpine treatment. Our secondary aims were to synthesise data pertaining to related symptoms (e.g. anxiety, suicide), overall tolerability and acceptability of reserpine treatment.

## Methods

The review adheres to the preferred reporting items for systematic reviews and meta-analyses (PRISMA) guidelines (Page et al., 2021). The review protocol was pre-registered on the international prospective register of systematic reviews (registration CRD42021225227).

### Search strategy

A systematic literature search was conducted of the electronic databases MEDLINE, Embase and PsycINFO (by RJ). The following search terms were used to identify publications from all dates up to 14 February 2021: (depress* OR MDD OR major depress* OR suicid* OR anxiety OR low mood) AND (reserpine OR serpasil). Reference lists from notable authors, review articles and articles eligible for inclusion were also hand searched for thorough data retrieval. ClinicalTrials.gov was also searched as part of the hand-searching process to identify unpublished trials. No language restrictions were implemented.

### Study eligibility criteria

Inclusivity of evidence was maximised to consider all possible effects of reserpine on depression. There were no restrictions on types of study design eligible for inclusion. Only primary studies of at least 10 participants were included. Participants had to be human adults, but no other restrictions were placed on participants’ age, gender, diagnosis or treatment.

Studies had to have assessed the effect of reserpine, in any dose and duration, on symptoms of depression. There must have been an untreated versus treated comparison, wherein data included adult participants not treated with reserpine, compared with those treated with reserpine. This could be either between-subjects (between treated and untreated participants) and/or within-subjects (between pre- and post-treatment time points). There was no restriction regarding comparator treatments. The primary outcome was differences in depressive symptoms between reserpine-untreated and reserpine-treated conditions. Studies which had no outcome for depression were excluded.

### Study selection and data extraction

Screening was conducted by two review authors (RJ and JC) independently assessing the search results against the pre-defined inclusion and exclusion criteria, blinded from one another’s selections. Any discrepancies between individual judgements were addressed by consensus through a third review author (RS or AHY). Upon agreement of included articles, data extraction was undertaken by two review authors (RJ and JC) independently, using a standardised extraction form. Data extracted pertained to study design characteristics, participant demographics and baseline characteristics (e.g. diagnoses), information regarding any between- or within-subjects characteristics, treatment characteristics (e.g. dose and duration of treatment), measures of outcome and relevant results. For the primary outcome, any assessment which captured depression was eligible, prioritising validated, clinician-rated measures of depression severity. Where this was not available, patient-rated validated depression measures, or non-standardised assessments of depression were considered. For secondary outcomes, data regarding extent of adherence to reserpine and any comparison interventions, such as trial dropout, non-completion, or other compliance data, were recorded. Additionally, extent of tolerability to reserpine was noted. This constituted data on side effects reported, highlighting those associated with mood or psychiatric symptoms, or discontinuation from the study for any reason. The effects of reserpine on other symptoms associated with depression, such as anxiety and suicide, or other individual symptoms of depression were also recorded.

### Quality assessment

The methodological quality assessment was examined using a modified risk of bias (RoB) assessment from the risk of bias in non-randomised studies of interventions (ROBINS-I) tool (Sterne et al., 2016). Due to the heterogeneity of study designs in this review, tailoring multiple RoB tools (combining or excluding some ROBINS-I items) was considered appropriate (Farrah et al., 2019). This was modified to enable assessment of non-longitudinal studies as described below. Studies were assessed by two independent reviewers for the following nine domains: sequence generation and allocation concealment (for randomised studies), comparability of intervention groups at baseline, blinding (participant and intervention), equal treatment of groups, use of intention-to-treat analysis, appropriateness of outcomes measured, deviations from protocol and allegiance effect. Each study was subsequently allocated an overall RoB rating of high, moderate or low RoB.

### Data analysis

Due to substantial methodological heterogeneity in study designs, populations studied and outcome measures employed, it was not statistically appropriate to conduct a meta-analysis in this review, as it may have obscured differences in effects or precluded a meaningful summary estimate of effect (Deeks et al., 2022). Therefore, a formal narrative synthesis on quantitative studies was conducted, in accordance with reporting guidelines of Synthesis Without Meta-analysis (Campbell et al., 2020), to strengthen the robustness of the narrative synthesis of results. Methodology and findings from the included articles are presented and analysed using a tabular method and narrative synthesis. To explore heterogeneity between studies, we considered potentially relevant subgroups or covarying factors comprised of study quality (RoB), diagnosis at baseline, duration and dose of reserpine. Additionally, based on data availability, we examined design (randomised, non-randomised, observational) and sample size of studies, and the type of depression measure (validated clinician rated, patient rated or non-validated) employed (Reeves et al., 2022). Where possible, pooled percentages for binary outcomes were calculated. Because studies varied greatly in how they measured and reported outcomes, their findings were synthesised using vote counting based on direction of effect (McKenzie and Brennan, 2022). Vote counting comprised comparing the number of effects showing a positive or negative association between reserpine and depression, with the number illustrating no association. The nature of the data did not allow us to assess the certainty of the synthesised findings. Secondary outcomes, namely tolerability, adherence and effects of reserpine on symptoms associated with depression were qualitatively explored.

### Changes made since protocol registration

Due to the inconsistency of methodologies and reporting of effect sizes between the included studies, we altered the measures of effect. Rather than using a standardised effect size, we employed vote counting as this was deemed more appropriate for syntheses of this nature (McKenzie and Brennan, 2022).

## Results

### Systematic search

**Figure 1.**
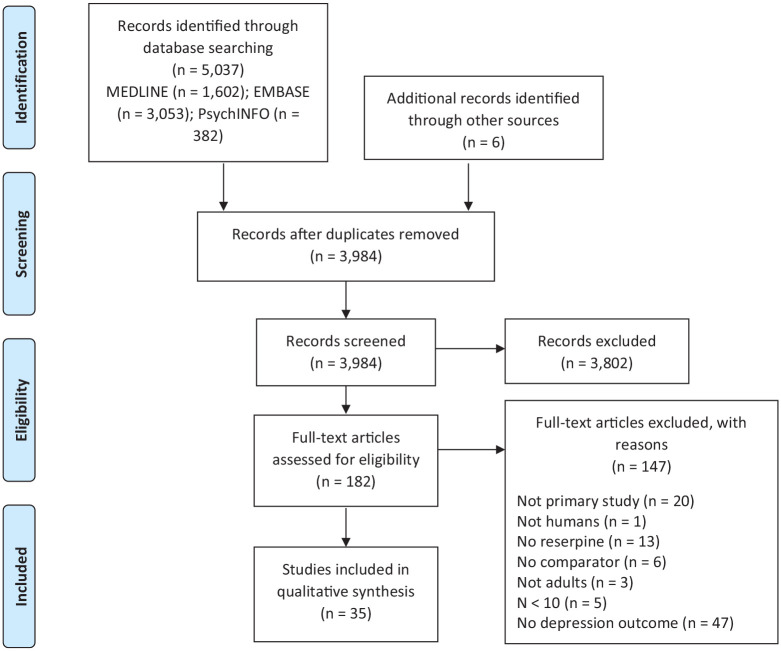
PRISMA flow chart. Flow chart depicting the literature screening process according to the PRISMA.

See Figure 1 for details of the search and study inclusion process. The systematic search yielded a total of 5037 records (3984 after removing duplicates). After an initial screen of eligibility based on article title and abstracts, 182 articles underwent a thorough full text review. Several articles were not accessible in a sufficiently detailed form to be considered (*n* = 52) or did not examine depression outcomes (*n* = 47); others were not primary studies (*n* = 20), did not administer reserpine (*n* = 13), did not have an untreated comparison (*n* = 6), did not include ⩾10 participants (*n* = 5) or were not of human adults (*n* = 4). Thirty-five articles were included in the review.

### Characteristics of included studies

Of 35 included studies, 16 (46%) were conducted in Europe, 15 (43%) in North America, 2 (5%) in Asia, 1 (3%) in South America and 1 (3%) in Australia. Their study designs varied: 17 (49%) were naturalistic studies, 9 (26%) randomised controlled trials, 6 (17%) non-randomised interventional studies and 3 (8%) cross-sectional comparisons. The cross-sectional studies were comparing depression outcomes in a group of patients who had been treated with reserpine with a matched control group who had no treatment or who were treated with a comparator drug. The median sample size was 42 (interquartile range = 56). The duration of treatment with reserpine ranged from 2 days (Carney et al., 1969; Hopkinson and Kenny, 1975) to 36 months (Bolte et al., 1959), with a median duration of 8 weeks (interquartile range = 22). Although most studies assessed reserpine alone (in addition to usual care), in one study, reserpine was augmented with imipramine, trimipramine and amitriptyline tricyclic antidepressants (Hopkinson and Kenny, 1975), one combined reserpine with imipramine (Carney et al., 1969), another with a ganglionic blocker (Platt and Sears, 1956). In four studies, patients were also given psychological intervention, that is, cognitive behavioural therapy or group psychotherapy (Bennett et al., 1956; Berger et al., 2005; Lowinger, 1957; Winhusen et al., 2007). The dose of reserpine ranged from 0.05 mg (Schwarz et al., 1973) to 130 mg daily (Sainz, 1955), administered orally or injected intramuscularly or intravenously. Seven studies were not written in English; for these, data were extracted by one native speaker (see acknowledgements) in addition to one English speaker using internet translation (RJ).

Table 1 provides summary characteristics of included studies; additional characteristics can be found in Supplemental Table 1. Participants had a mean age of 48 years (s.d. = 11, range 26–70) and on average 55% were female. The population assessed varied between studies: 15 (43%) studied participants with hypertension, 9 (25%) included various psychiatric conditions, 4 (11%) had a depression population, 2 (6%) involved cocaine dependent participants, 2 (6%) had a schizophrenia population, 1 (3%) included anxiety, 1 (3%) used healthy volunteers and 1 (3%) included anxiety and depression.

**Table 1.** Characteristics of included studies.

Reference	Population	Comparator group(s)	*N* participants	Reserpine duration (weeks)	Reserpine dose (mg)	% depressed (baseline)	Primary outcome measure
Interventional studies							
Davies et al. (1955)	Anxiety and depression	Placebo	28	6	1	100	Clinician assessment
Berger et al. (2005)	Cocaine dependence	Gabapentin, lamotrigine, placebo	15	8	0.5	NA	HAM-D
Hopkinson et al. (1975)	Depression	Placebo	8	2 days	5 IM	100	HAM-D
Veselinović et al. (2011)	Healthy volunteers	Aripiprazole, haloperidol, placebo	18	1	0.25–1	NA	HAM-D
Winhusen et al. (2007)	Cocaine dependence	Placebo	42	12	0.25–0.5	NA	HAM-D
Hodgkinson *(1956)*^a^	Hypertension	Placebo	35	52–78	1–2	NA	Clinician assessment
Wachspress et al. (1956)	Various psychiatric	Placebo	15	12	5–10 (IM/oral)	40	Clinician assessment
Finn et al. (1955)^a^	Schizophrenia	Placebo	22	12	1.5–15	NA	MSRPP
Azima et al. (1959)	Schizophrenia	Placebo	10	4	3–10	NA	Clinician assessment
Achor et al. (1955)^a^	Hypertension	Whole-root preparation, placebo	58	8	0.4	NA	Clinician assessment
Platt et al. (1956)^a^	Hypertension	Placebo	54	Variable	0.5–2	NA	Clinician assessment
Santucci et al. (1989)	Hypertension	Captopril, metoprolol, methyldopa	40	12	0.125	NA	Clinician assessment
Fife et al. *(1959)*^a^	Hypertension	Placebo	71	6^d^	0.75–1.5	NA	Clinician assessment
Segal et al. (1959)	Anxiety^b^	Placebo	42	3	1	NA	Clinician assessment
Lowinger (1957)^a^	Various psychiatric	Crude root extract, AF	70	3	0.75–14	23	Clinician assessment
Naturalistic studies							
Bolte et al. (1959)	Hypertension	Crude root extract, AF	270	4–156	<0.5–>0.75	NA	Clinician assessment
Wallace (1955)	Hypertension	Various^c^	44	52	NR	NA	Observation
Sainz (1955)	Depression^b^	NA	41	26	3–130 IV/oral	100	Clinician assessment
Lemieux et al. (1956)	Hypertension	Whole root extract, AF	134	78	0.75–4	NA	Clinician assessment
Vakil (1949)	Hypertension	NA	50	6	NR	NA	Patient report
Drake et al. (1955)	Various psychiatric	NA	40	1–26	0.125–4	NA	Clinician assessment
Kirkegaard et al. (1958)	Various psychiatric^b^	NA	1027	Variable	1–20 (IM/oral)	13	Clinician assessment
Bennett et al. (1956)	Various psychiatric^b^	Chlorpromazine	91	Variable	3/5 oral/IM	38	Clinician assessment
Hiob et al. (1955)	Various psychiatric^b^	NA	55	6	6–15 mg (avg 9.2)	47	Observation
Pellerito (1956)	Various psychiatric	Chlorpromazine	~100	Variable	⩽3	NR	Observation
Krajnáková et al. (1981)	Hypertension	*Rauwolfia crystepin*	36	Long term	NR	NA	BDI
Schwarz et al. (1973)	Hypertension	Clonidine, placebo	80	4	0.05–0.5	NR	HAM-D
Ingrova et al. (1963)	Various psychiatric	NA	24	4	3–6 (IM/oral)	22	Observation
Jeri (1957)	Psychosis	NA	159	4–6	10–30 (IM and oral)	0	Observation
Kirk et al. (1970)	Depression^b^	NA	24	Single dose	10 IM	100	Clinician assessment
Carney et al. (1969)	Depression	NA	8	2 days	10	100	HAM-D
Bant (1978)	Hypertension	Diuretic, methyldopa, adrenergic/beta-blockers	20	52	0.2	NA	BHPT (score > 40)
X-sec							
Zhu et al. (2019)	Hypertension	NA	787	52+	0.1–0.2	NA	ZSDS (score > 52)
Prisant et al. (1991)	Hypertension	Diuretic, beta-blockers, no drug	111	13+	<0.125–>0.125	NA	ZSDS ⩾ 50
Dissegna et al. (1985)	Hypertension	Beta-blockers, methyldopa, clonidine, diuretic	73	NR	0.1–0.25	NA	Kellner and Sheffield

AF: alseroxylon fraction; BDI: Beck’s depression inventory; BHPT: British Hospital Progress Test; HAM-D: Hamilton Depression Rating Scale; IM: intramuscular, IV: intravenous; MSRPP: Multidimensional Scale for Rating Psychiatric Patients; NA: not applicable; NR: not reported; X-sec: cross-sectional study; ZSDS: Zung self-rating depression scale.

a Non-randomised.

b Describes eligible depression subgroup where full sample did not meet review inclusion criteria.

c Phenobaritone, aminophyllin, ganglion-blockers, parenteral hexamethonium, combined reserpine and pentolinium.

d Outcomes came from follow-up (follow-up period average of 13.4 months).

### Quality assessment

Supplemental Table 2 displays the RoB ratings across criteria and studies. The majority of studies were undertaken prior to the introduction of clear research conduct and reporting standards and were rated to have a high RoB, with only two rated as low RoB and three as moderate RoB. Each of the low or moderate RoB studies appeared to have higher quality controls than other studies in terms of being randomised, with a placebo condition and a clinician-rated validated depression outcome (although were not necessarily more recent or recruiting an adequate sample size). Notably, there was no evidence of pre-specified methods or outcomes in all but one study (Zhu et al., 2019). Only one study (Veselinović et al., 2011) was indicated to have a potential conflict of interest through being affiliated with an industrial sponsor.

**Table 2.** Depression outcome findings.

Reference	*N*	Treated depression severity		% depression		Adherence (%)	Discontinuation (%)	Notable findings
		Reserpine	Comparator (s)	Reserpine	Comparator (s)			
A. Reserpine untreated versus treated groups								
Zhu et al. (2019)	787	40	41	12	12	NA	NR	No between-group difference
Hopkinson et al. (1975)	8	11	22	NR	NR	100	0	Depression reduced (from 30 reserpine/28 placebo)*
Bolte et al. (1959)	270	51/6^a^	8/12	39	32/33	NR	NA	High > low dose^#^
Bant (1978)	20	42	41–56	60	39–55	70 (vs 63–94)	30 (vs 6–38)	No between-group difference
Krajnáková et al. (1981)^b^	36	NR	NR	39	60	NR	NR	Within-subjects depression increase for crystepin and reserpine^#^
Davies et al. (1955)	28	NR	NR	29^e^	58^e^	88 (vs 93)	29	Reserpine < placebo*
Prisant et al. (1991)	111	44–46	45–47	35	40	NR	NR	No between-group difference
Lemieux et al. (1956)^b^	134	NR	NR	18	8	NR	NR	Psychosis *n* = 2, dose reduce improved depression *n* = 5^#^
Winhusen et al. (2007)	42	2.11	3.18	NR	NR	79	30 (vs 37)	Depression reduced (from severity score 4 both groups)**
Santucci et al. (1989)	40	NR (64% depressed at baseline)		63	50–74	NR	NR	No between-/within-group difference^#,c^
Hodgkinson (1956)	35	NR	NR	3	0	NR	3	*n* = 1 extreme depression^#^
Platt et al. (1956)	54	NR	NR	19	0	NR	19	Suicide *n* = 1 reserpine and within-subjects depression increase^#^
Wallace (1955)	44	NR	NR	9	14	NR	2	Depression higher only when combined with pentolinium^#^
Achor et al. (1955)^b^	58	NR	NR	10	16	NR	9	Within-subjects depression increase for whole root and reserpine^#^
Segal et al. (1959)	42	NR	NR	31% affective complaints to either arm		NR	41	No between-group difference
Dissegna et al. (1985)	73	3	3	NR	NR	NR	NR	No between-group difference
Pellerito (1956)	~100	NR	NR	68^c^	50^d^	NR	NR	Within-subjects therapeutic effect, reserpine < chlorpromazine^#^
Veselinović et al. (2011)	18	3.3	0.2–1.4	56	6–33	100	0% (vs 0–39)	Depression rose in all groups, most on reserpine*
Bennett et al. (1956)	91	NR: 38% depressed at baseline; neither group showed depression change				NR	NR	No between/within-group change^#^
Schwarz et al. (1973)	80	NR	NR	10	0	NR	NR	Within-subjects depression decrease, reserpine < others^#^
Reference		*N*		Untreated severity	% depression treated	Adherence (%)	Discontinuation (%)	Notable findings
B. Reserpine untreated versus treated time points								
Sainz (1955)		41		(all depressed)	32	NR	NR	22/41 remitted under reserpine
Azima et al. (1959)		10		(none depressed)	40	100	0	Mania *n* = 1, but 90% elation; 40% mixed state
Lowinger (1957)		70		23% untreated depressed; no change on reserpine		NR	NR	Depression not affected
Berger et al. (2005)		15		HAMD score 7 untreated to 10 treated		30 (vs 80/90)	27 (vs 20 vs 14 vs 6)	HAMD NS change; self-rated depression (BDI) improved
Vakil (1949)		50		(none depressed)	8	variable	6	No serious reactions reported
Wachspress et al. (1956)		15		33% untreated depressed; 27%worsened on reserpine		NR	13	Worsened on reserpine. Suicidal ideation *n* = 1
Fife et al. (1959)		71		(none depressed)	21	NR	38	Reserpine dose reduced due to high depression
Drake et al. (1955)		40		(43% depressed)	76% unchanged	NR	NR	Suicide attempt *n* = 1 reserpine
Kirkegaard et al. (1958)		1027		(13% depressed)	[see end column]	NR	55	7/183 depression worsened; 128/183 improved
Hiob et al. (1955)		55		(52% depressed)	18	NR	NR	Reserpine improved depressed patients but dysphoria observed in other illnesses
Kirk et al. (1970)		24		(all depressed)	8% worsened	100	0	10 no change; 9 slightly improved; 3 greatly improved
Carney et al. (1969)		8		Severity reduced from 18 to 10	38% unchanged	NR	20	Mania *n* = 1 reserpine. Depression reduced slightly (*p* < 0.05)
Finn et al. (1955)		22		(‘severely disturbed’ inpatients)	5% worsened	100	0	18/22 improved; 9 marked improvement; 3 no improvement; 1 worsened
Ingrova et al. (1963)		24		(92% depressed)	41	NR	NR	0 negative, 20 positive, 2 no change, 1 hypomania
Jeri (1957)		159		0	6	NR	NR	Worsened on reserpine

A: Where possible, between-subjects associations of depression between reserpine and other conditions (note that in some cases, within-subjects indications are also noted in the final column). B: Where no between-subjects data are available, within-subjects reports between untreated and treated conditions with reserpine. ‘severity’ data is presented as averages and rates (%) are presented for binary outcomes on people/times untreated or treated with reserpine. mg: milligram; NA: not applicable; NR: not reported; NS: non-significant.

a *n* depressive episodes.

b Comparator also contained rauwolfia.

c However, % of good quality of life increased from 44% to 55% after reserpine

d % without ‘therapeutic mood response’

e U﻿nchanged or worsened

* Within- and between-subjects comparisons both significant (in some cases a within-subjects statistical comparison was not undertaken but significance was inferred by large effects reported).

** Within-subjects but not between-subjects statistically significant.

# Statistical significance not inferable (usually due to absence of statistical comparisons conducted).

### Primary outcome

Depression rates after reserpine treatment ranged from 3% (Hodgkinson, 1956) to 76% (Drake and Ebaugh, 1955) of participants across different study types. Table 2 displays these findings across studies. Notably, about half of the studies included a psychiatric population at baseline and non-psychiatric studies did not always exclude people with psychiatric symptoms prior to reserpine treatment.

#### Between-subjects effects

As presented in Table 2, of the 20 studies (*n* = 2071 participants) with a between-subjects comparison, numerically higher depression (severity scores or episode rates) was reported in reserpine-treated patients than in untreated patients in eight studies (*n* = 711 participants); no difference between treated and untreated (or other-treated) groups was reported in six studies (*n* = 1077) and lower depression in six studies (*n* = 283).

Where reserpine was associated with depression, this was reported as statistically significant in one study, compared with aripiprazole, haloperidol and placebo (Veselinović et al., 2011). Rates were numerically higher (statistical tests not undertaken) in comparison to crude root extract and asteroxyon fraction (Bolte et al., 1959; Lemieux et al., 1956), placebo (Platt and Sears, 1956), clonidine and placebo (Schwarz et al., 1973). The difference was smaller in one study compared to chlorpromazine (Pellerito, 1956) and another to placebo (Hodgkinson, 1956) and in only one of these studies with a statistical comparison, non-significantly higher than diuretic, methyldopa, adrenergic blockers or beta-blockers (Bant, 1978).

Clearer no-difference effects were in comparison with chlorpromazine (Bennett et al., 1956), placebo (Segal and Shapiro, 1959), beta-blockers, methyldopa, clonidine, diuretic (Dissegna et al., 1985) and other-treated participants in a cross-sectional examination (Zhu et al., 2019). Additionally, one study reported no significant difference in reserpine monotherapy compared with a variety of other medications (although overall, depression was numerically lower than comparators), except for a higher rate of depression identified in participants treated with both reserpine and pentolinium than other comparators (Wallace, 1955); a final study identified (numerically) lower rates of depression in reserpine than alpha methyldopa but higher depression in reserpine than in either captopril or metoprolol (Santucci et al., 1989).

Finally, of the six studies finding less depression in reserpine-treated individuals, two were numerically lower (not statistically) than diuretic, beta-blockers, placebo (Prisant et al., 1991) and another versus placebo (Winhusen et al., 2007). Others, not tested statistically, were compared with whole-root extract and placebo (Achor et al., 1955) and crystepin (Krajnáková et al., 1981). However, both are also derived from *R. serpentina*. Finally, statistically lower depression was reported in two placebo-controlled studies (Davies and Shepherd, 1955; Hopkinson and Kenny, 1975).

Despite these between-group effects, some of the studies where between-groups comparisons did not find reserpine to elicit higher depression did identify within-group worsening with reserpine (Achor et al., 1955; Krajnáková et al., 1981) and in others where more depression was reported in reserpine than other groups, within-subjects improvements even in reserpine groups were reported (Schwarz et al., 1973).

#### Within-subjects effects

Twenty-seven studies provided evidence of changes in depression over time (*n* = 2320 participants). As above, most did not report statistical significance for within-subjects effects; therefore results are reported in terms of *numerical* direction. Eleven studies (*n* = 645) found some increase in depression under reserpine; 4 studies (*n* = 216) found no changes in depression and 12 studies (*n* = 1459) reported some reduction in depression following reserpine therapy. Most notably, one study found that 54% depressed individuals remitted under reserpine (Sainz, 1955); another study from the latter category did report an improvement in depressive illness for those with symptoms prior to treatment but dysphoria emerging in other patient groups (Hiob and Hippius, 1955). One study recorded above as ‘no change’ reported a slight (non-significant) increase in clinician-rated depression severity scores, but a larger improvement in patient-rated scores after reserpine (Berger et al., 2005). Two other ‘no change’ studies reported that despite a lack of overall improvement, reserpine had helped patients to engage with other concomitant antidepressant therapies and that it could be a useful adjunctive agent from this perspective (Bennett et al., 1956; Lowinger, 1957).

Relatedly, across within- and between-subjects findings, it is relevant that three of the studies finding no association and three reporting improvement were concomitantly treating patients with an antidepressant or psychotherapy (studies referenced above).

When pooling depression rates after reserpine across all available studies, the rate was 27% for participants with psychiatric illnesses at baseline (19 studies, *n* = 1821; rate not reported in an additional four studies) while the rate for non-psychiatric patients was 23% (16 studies, *n* = 1881; rate not reported in one study). What differed more between these participant groups was the rate of depression in placebo-treated participants; 10% across eight psychiatric population studies (*n* = 182) and 1% from six non-psychiatric studies (*n* = 316). If then calculating the percentage difference overall between participants in reserpine compared with control arms, an increase in depression of 17% is found in populations with mental illnesses (27% reserpine vs 10% placebo) and the increased rate of depression in populations without mental illnesses is 22% (23% reserpine vs 1% placebo). Speculatively, this could support the view that reserpine is less depressogenic in people with existing psychiatric illnesses.

### Primary outcome effect modifiers

We considered potential effect modifiers by categorising all studies into three categories: *Negative studies*, where their findings overall suggested a significant or possible depressogenic effect of reserpine (11 studies); *No-effect studies*, where their findings did not demonstrate an effect (13 studies) and *Positive studies*, where a potential therapeutic effect on depression was suggested (11 studies). See Table 3 for a summary of the putative effect modifiers across each category, and Supplemental Table 3 contains a more detailed breakdown of all these findings.

**Table 3.** Summary of subgroup influences on results by category.

Category	Potentially antidepressant (‘positive’ studies)	No depression effect (‘no-effect’ studies)	Potentially depressant (‘negative’ studies)
	*n* = 11	*n* = 13	*n* = 11
Participant population	*Psychiatric populations*	Non-psychiatric populations	Non-psychiatric populations
% depressed (in reserpine-untreated condition)	*Some/all participants depressed*	None depressed	None depressed
Study design	Non-randomised interventional	Non-randomised interventional	Non-randomised interventional
Sample size	Small sample size	*Small–medium sample size*	*Small–medium sample size*
Reserpine duration	*Short/moderate duration*	*Short/moderate duration*	–
Reserpine dose	*Not low dose*	Low dose	Variable/moderate dose
Depression measure	Non-validated assessment	Non-validated assessment	Non-validated assessment
RoB level	High RoB	High RoB	High RoB

Summary indication of subgroup effect indicated by the majority (⩽50%) of studies (excluding those where the data was not reported) within one of the three categories sharing a characteristics. The full results at a study level are presented in Supplemental Table 3. Italic text refers to the merging of two groups of characteristics similar in nature. “–” is given where no majority effect is evident; one example here is that 27% of negative studies were long durations, 27% moderate duration, 27% low and 18% variable.

*Pre-existing mental illness*: Positive studies were more likely to examine (not depression-specific) psychiatric populations, and none recruited non-psychiatric patients; non-psychiatric patient samples were more frequently examined in no-effect and negative studies. Some studies found that depressogenic effects of reserpine were less likely within depressed participants (Hiob and Hippius, 1955) and therefore the proportion of (reserpine-untreated) participants who were classified as depressed was considered as a separate potential effect modifier.

*Baseline depression:* 10/11 (91%) negative studies examined participants who were not depressed before starting treatment and this was also the case for no-effect studies. By contrast, 7/11 (64%) of positive studies included some/all participants with depression at baseline.

*Depression outcome measure:* Most studies employed a non-validated measure of depression; clinician-rated validated measures were slightly more frequent in the positive studies, and no-effect studies comprised the majority of patient-rated assessment.

*Dose:* Negative studies frequently used moderate and/or variable doses, whereas no-effect studies frequently used low doses, and positive studies were slightly less likely to use low doses. Notably, one negative and one no-effect study both reported higher rates of depression in participants taking a higher dose of reserpine (Bolte et al., 1959; Prisant et al., 1991) and others have reported lowering doses of reserpine as a direct result of depressive symptoms emerging (Lemieux et al., 1956).

*Duration:* No positive studies treated patients with reserpine for longer than 6 months (studies were equally distributed between short or moderate durations). Duration was more evenly distributed across categories for no-effect and negative studies (with long durations in 31% and 27% of studies, respectively).

*Design:* Naturalistic studies were well represented in all three categories, but no-effect studies were less likely to be randomised trials, and negative studies were slightly more likely to be non-randomised interventional designs.

*Sample size:* Positive studies were less likely to examine large samples (9%; 73% small samples). The other two groups were more evenly distributed between categories, although negative studies were more often either large or small than no-effect studies (more often medium).

Newer studies did not consistently assess larger samples than older studies, though one of the four studies published in this century was large and was classified as no effect. The other three newer studies were categorised as positive (two studies) or negative (one study). Two out of four newer studies comprised 2/5 of the low/moderate RoB studies (one was a positive study, the other was negative and both had small sample sizes).

*Risk of bias:* As most studies were categorised as having a high RoB, we report here only the classification of the five low/moderate RoB studies: 3/5 were positive and 2/5 were negative (all small samples).

### Secondary outcomes

#### Adherence

These data are included in Table 2. Two-thirds of studies did not report adherence or discontinuation of treatment with reserpine or comparators. Across arms, the highest dropout rate was observed in a haloperidol comparator (39%; (Veselinović et al., 2011)) in contrast with a reserpine discontinuation of 0%. There was a difference of >10% in participant discontinuation between arms in Bant (1978) (17% antihypertensive drugs; 36% non-antihypertensive comparisons) and Berger et al., (2005) (27% reserpine; 7% lamotrigine; 7% placebo; 0% gabapentin). Ten studies reported adherence data, which was numerically lower in reserpine versus active and placebo controls in three studies (Bant, 1978; ; Berger et al., 2005; Davies and Shepherd, 1955).

#### Tolerability and related symptoms

Seventeen studies reported an average proportion of the sample reporting adverse events. These were higher in reserpine than in some comparators (active/placebo) in six studies (Bant, 1978; Davies and Shepherd, 1955; Fife et al., 1959; Hodgkinson, 1956; Hopkinson and Kenny, 1975; Lowinger, 1957), although two of these studies were higher in other rauwolfia formulations than in reserpine or placebo (Achor et al., 1955; Santucci et al., 1989). The most common side effect reported was sedation, although one manic onset was also reported in two studies (Azima et al., 1959; Carney et al., 1969). One further study noted a psychotic reaction in two patients following reserpine (Lemieux et al., 1956).

Increased anxiety was reported in six studies (see Supplemental Table 1) and suicide or suicide attempt or suicidal ideation reported in three studies (see Table 2). There were no suicidal trends reported in comparator arms. Individual symptoms of depression were reported as a side effect to reserpine treatment; fatigue was reported in 10 studies (Bennett et al., 1956; Fife et al., 1959; Finn et al., 1955; Hodgkinson, 1956; Kirkegaard et al., 1958; Lemieux et al., 1956; Sainz, 1955; Vakil, 1949; Veselinović et al., 2011; Wachspress et al., 1956). Concentration deficits were reported in Veselinović et al. (2011); insomnia and sleep disturbances were reported in Fife et al. (1959), Vakil (1949), Pellerito (1956) and Veselinović et al. (2011). Loss of motivation, interest or drive were reported in Veselinović et al. (2011) and Hiob and Hippius (1955). Lemieux et al. (1956) reported decreased appetite, while Davies and Shepherd (1955), Jeri (1957) and Wachspress et al. (1956) reported an increase in appetite. Reserpine was associated with weight gain in six studies (Achor et al., 1955; Fife et al., 1959; Finn et al., 1955; Jeri, 1957; Vakil, 1949; Wachspress et al., 1956).

## Discussion

This systematic review is, to our knowledge, the first to evaluate the effects of reserpine on depressive symptoms. Our findings highlight the limited evidence base, with few adequately powered controlled studies, high heterogeneity between studies and a high RoB.

The prevalence of depression following reserpine treatment ranged from 3% to 87% (although studies with higher depression symptoms post-reserpine also included depressed individuals in the untreated condition). Eleven studies reported depressive effects of reserpine, 13 reported an absence of effect and 11 reported potential benefits for depression symptoms with reserpine. Studies suggestive of a depressive effect were more likely to examine non-psychiatric patients at baseline, treat participants for longer and at lower doses compared with studies suggestive of an antidepressant effect. Despite indications that reserpine’s inhibition of VMAT function (explaining its widespread non-selective depletion of monoamines) could be dose and duration dependent (Mahata et al., 1996), its pharmacological effects have not been fully characterised. Thus, we are not able to attribute any potential impact of variation in interventional (e.g. dose/duration) factors’ influences on depression to biological effects.

### Exploring heterogeneity

These conflicting findings reflect inconsistencies in the literature, from early case studies on reserpine (Freis, 1954) to more recent arguments challenging these concerns (Healy and Savage, 1998). Because our systematic review was inclusive, the studies included would inevitably be highly variable in terms of methodology and quality. This was the case across a range of domains, including study design (from cross-sectional comparisons to naturalistic studies, randomised and non-randomised trials); RoB; date of publication; sample size; participant factors (most notably, psychiatric status prior to initiating reserpine); treatment factors (e.g. dose and duration) and outcome factors (e.g. measures used to assess depression). We examined these as subgroups to determine their relationship with study findings. Most of these subgroups did not show a definitive association with depression findings, although a therapeutic effect was observed only in studies recruiting psychiatric patients and none of these studies examined durations of reserpine treatment exceeding 6 months. Some studies treated participants particularly for a short duration, as little as 2–4 days in two studies that observed reductions in depression severity after reserpine (Carney et al., 1969; Hopkinson and Kenny, 1975). Early work had suggested that at least 6 weeks were needed to observe the emergence of depression after reserpine (Nick, 1955).

Almost two-thirds of included studies used a non-validated assessment of depression, usually clinician judgement or observation, and a further 14% used patient-report assessments. Both can generate false-positive cases, which would overestimate clinically significant depression switches after reserpine (Leon et al., 1997) and this is supported by Baumeister et al.’s (2003) non-systematic review findings. This leaves only seven studies evaluating depression using a potentially valid method (diagnostically), of which four had reported putative antidepressant effects.

Given the number of combinations of these factors alongside the small number of articles and patients studied, we cannot make conclusions regarding the circumstances under which reserpine causes or treats depressive symptoms. What we can conclude, though, is that the story of reserpine is not as straightforward as has been widely assumed; thus this work extends previous literature by highlighting that the broadly accepted notion that reserpine should not be used requires further investigation.

We did not assess all possible covarying factors, for example the age of participants: the prevalence of depression reported after reserpine treatment was approximate to the upper limit of point prevalence estimates for depression in the general population (using self-report to assess depression (Lim et al., 2018), and rates of depression may be higher in older people (Kim et al., 2020)). Many samples in our review were older hypertensive patients, and therefore rates of depression in these people may well be relatively large, though not necessarily larger than the general population of people in this age range.

### Putative antidepressant effects of reserpine?

The 11 studies identifying potential therapeutic effects in our review align with a previous placebo-controlled investigation of reserpine for patients with treatment-resistant depression (*n* = 9; ineligible for this review) finding that reserpine produced rapid improvements when adjunctive to tricyclic antidepressants. However, treatment only lasted for a short period of 7 days (Amsterdam and Berwish, 1987). Taken together, this supports the view that reserpine might be an antidepressant and underpins a potential use for reserpine in the treatment of depression when combined with an antidepressant or psychotherapy.

Some of the studies reporting reserpine’s benefits on depression administered concomitant antidepressant therapies, for example, tricyclic antidepressants or psychological input. Many found that reserpine improved the engagement or efficacy with other treatments, but of the 11 positive studies, two were delivering reserpine monotherapy (Kirk et al., 1970; Pellerito, 1956) and five treated reserpine only in addition to various (non-psychotropic) continuation treatments (Davies and Shepherd, 1955; Hiob and Hippius, 1955; Ingrova et al., 1963; Kirkegaard et al., 1958; Sainz, 1955).

Additionally, the study reporting the highest prevalence of post-reserpine depression (87%) was in a mixed psychiatric population of whom 38% were depressed before reserpine initiation (Drake and Ebaugh, 1955). This suggests reserpine can be depressogenic in those susceptible to the illness (Upthegrove et al., 2017) and questions the idea that reserpine may be an antidepressant in those with pre-existing clinically significant symptoms, although this was one of the longer studies, treating patients for 6 months.

Conversely, the overall pooled percentages for depression across our included studies indicated a 3% difference in depression onset after reserpine treatment between psychiatric patients and non-psychiatric patients, whereas the risk for depression after placebo was 7% higher in psychiatric versus non-psychiatric participants. One possible explanation for this relates to a possible misdiagnosis (in some cases) of depression instead of akathisia (Healy and Savage, 1998) as akathisic reactions may emerge as an adverse effect more frequently in non-psychiatric populations (Healy and Farquhar, 1998) and therefore may explain the negative effects observed in samples with hypertension.

### Depression-associated symptoms

Even if the psychiatric adverse effects are questionable, the consequences are significant. Previous research reported one suicide and three attempted suicides during 1 year’s treatment with reserpine in patients with schizophrenia (Jorstad, 1956) corroborating findings of three of our included studies (Drake and Ebaugh, 1955; Platt and Sears, 1956; Wachspress et al., 1956) and a previous theory that reserpine-induced depression is characterised by suicidal thoughts, albeit from a case study of six patients (Nordman, 1956). The quantity of evidence is not sufficient to disregard the possibility of this suicidality being attributable to natural courses of psychiatric illness. Relatedly, there remain uncertainties about risk of manic switch after reserpine (Azima et al., 1959; Carney et al., 1969). The effect of reserpine on anxiety is questionable, as four of our studies found anxiogenic effects (some on patients with pre-existing anxiety) (Peterfy et al., 1976) while others reported anxiolytic effects potentially via sedation (Hiob and Hippius, 1955; Hodgkinson, 1956).

Some articles reported increases in other individual symptoms of depression, for example reduction in concentration (Veselinović et al., 2011), or anorexia (Vakil, 1949). These are non-specific symptoms though, and if documented in the absence of the core elements of depression (low mood, anhedonia), may inflate estimated depression rates (Moulton et al., 2021).

### Relevance for mechanisms underlying antidepressant response and monoamines

Our findings pose a challenge to the original monoamine hypothesis of depression which has largely dominated the field, as reserpine acts by depleting catecholamines (Cheung and Parmar, 2022), yet has been evidenced to improve depressive symptoms in some cases. The putative depressogenic effect of reserpine has been largely cited in support of the basic monoamine hypothesis (Carlsson, 2001). Findings of some antidepressant properties of reserpine contradict this (Healy and Savage, 1998), supplementing other literature that has identified no depressive effect from manipulating tryptophan (Ruhé et al., 2007). This may also have implications for the reserpine animal model which is still used as a pharmacological challenge for putative antidepressants (El-Marasy et al., 2021), though it is acknowledged that animal models of depression are difficult to extrapolate to the complex behavioural phenotypes of depression. No studies included in this review examined biological markers before and after reserpine treatment. However, because almost half of included studies did report some depressogenic effects of reserpine, it is possible that a subset of people could be susceptible to reserpine-induced depression based on, for example, catecholamine activity. This is as yet an unsubstantiated speculation.

## Strengths and limitations

Although the studies included in this review were highly heterogeneous (reflected in inconsistent findings between studies) and date back in many cases to the 1950s, we posit that this review was strengthened by its inclusive approach and adds to the extant literature as the first systematic examination of reserpine’s effects on depression. The small number and size of studies, their high RoB and variable designs limit conclusions that can be drawn and precluded a quantitative meta-analysis. We investigated a range of relevant potential effect modifiers to interpret our findings, although the quantity and heterogeneity (e.g. of population, dose, duration, methodology) limit interpretations that can be drawn. It is also worth acknowledging that some of the older studies had a low RoB.

Moreover, our RoB ratings are undertaken according to current research practices, one example being the pre-registration of protocols to ensure rigour and transparency of analysis and findings (Al-Jundi and Sakka, 2016). In this respect and others (e.g. many older studies did not conduct statistical analyses of depression emergence) the reviewed literature is outdated and new studies recruiting adequate sample sizes and employing bias minimisation strategies are required. Although we made maximum efforts to obtain all available data, there were articles that could not be accessed and authors we could not contact for more detailed data.

## Conclusions

There has been long-standing controversy surrounding the notion that reserpine causes depression. This review has not uncovered conclusive evidence to elucidate the role reserpine has in inducing – or treating – depressive symptoms. However, it represents the first systematic consolidation of this literature, and we propose enhancing our present understanding of the effect of reserpine on depressive symptoms in humans. Given that the studies which have reported depressogenic effects of reserpine tended not to be randomised trials, administered reserpine for longer and at lower doses and were more likely to examine non-psychiatric patients, we call for rigorous controlled clinical studies to examine the outcomes of time-limited reserpine at a moderate dose with standardised mood stabilising or antidepressant therapies. Of equal importance, we urge a balanced and careful judgement regarding reserpine’s effects as being complex and multifaceted.

While the clinical studies of reserpine appear equivocal, the results of this review do cast doubt on simplistic notions underlying the initial monoamine hypothesis of depression. Wide individual variation in response of depression symptoms to this drug suggests a more nuanced approach is necessary when evaluating the effects of catecholamine depletion on depression syndromes.

## Supplemental Material

sj-docx-1-jop-10.1177_02698811221115762 – Supplemental material for The effects of reserpine on depression: A systematic review Click here for additional data file. Supplemental material, sj-docx-1-jop-10.1177_02698811221115762 for The effects of reserpine on depression: A systematic review by Rebecca Strawbridge, Rahila R Javed, Jeremy Cave, Sameer Jauhar and Allan H Young in Journal of Psychopharmacology
