# Supplementary material for: The effects of reserpine on depression: A systematic review
Source: J Psychopharmacol. 2022 Aug 24;37(3):248–60. doi: 10.1177/02698811221115762 (PMC10076328; doi:10.1177/02698811221115762)
Supplement: sj-docx-1-jop-10.1177_02698811221115762 – Supplemental material for The effects of reserpine on depression: A systematic review [file sj-docx-1-jop-10.1177_02698811221115762.docx]

**Supplementary material**

*Supplementary table 1: additional characteristics of included studies*

|  | Reference | Continent of study | Setting | n | Average age | % female | Anxiety outcome |
| --- | --- | --- | --- | --- | --- | --- | --- |
| **Interventional studies** | Davies et al., 1955 | Europe | In/outpatient | 28 | 38 | 50 | 32% unimproved; 68% improved after reserpine |
|  | Berger et al., 2005 | N America | Outpatient | 15 | 39 | 30 | HAM-A^f^ scores rose 3.7 - 6.6 (reported statistically significant but not clinically significant rise in anxiety) |
|  | Hopkinson et al., 1975 | N America | Inpatient | 8 | 33 | 100 | - |
|  | Veselinović et al., 2011 | Europe | Community | 18 | 26 | 53 | 33% reported anxiety side effect |
|  | Winhusen et al., 2007 | N America | Community | 42 | 41 | 28 | - |
|  | Hodgkinson, 1955 | Europe | Outpatient | 35 | NR | NR | - |
|  | Wachspress et al., 1956 | N America | Inpatient | 15 | 33 | 53 | Anxiety increased 6/17; reduced 3/17; no change 8/17 |
|  | Finn et al., 1955 | N America | Inpatient | 22 | 38 | 0 | Reduction in anxiety and tension |
|  | Azima et al., 1959 | N America | Inpatient | 10 | 45 | 0 | 40% anxiety after reserpine |
|  | Achor et al., 1955 | N America | Outpatient | 58 | 57 | 74 | - |
|  | Platt et al., 1956 | Europe | Outpatient | 54 | NR | NR | - |
|  | Santucci et al., 1989 | Europe | Outpatient | 112 | NR | NR | *Absence* of anxiety rose from 11-22% after reserpine |
|  | Fife et al., 1958 | Europe | Outpatient | 71 | NR | 63 | - |
|  | Segal et al., 1959 | N America | Outpatient | 42 | NR | 36 | 23/41 anxiety improved; 19/41 anxiety unimproved |
|  | Lowinger, 1957^e^ | N America | In/outpatient | 70 | NR | NR | 21% no change or reduced anxiety |
| **Naturalistic studies** | Bolte et al., 1959 | N America | Outpatient | 270 | 50 | 74 | - |
|  | Wallace, 1955 | Australia | Outpatient | 44 | NR | NR | 1/44 anxiety after reserpine; 1/44 no change anxiety |
|  | Sainz, 1955 | N America | In/outpatient | 41 | NR | 58 | Anxiety decreased after reserpine |
|  | Lemieux et al., 1956 | N America | Outpatient | 134 | 53 | NR | 7% anxiety after reserpine |
|  | Jal Vakil, 1949 | Asia | Outpatient | 50 | 59 | 40 | - |
|  | Drake et al., 1955 | N America | Outpatient | 40 | NR | NR | Reserpine improved 8/9 anxiety patients * |
|  | Kirkegaard et al., 1958 | Europe | Inpatient | 1027 | NR | 62 | improvement in 11 out of 23 anxiety cases * |
|  | Bennett et al., 1956 | N America | In/outpatient | 91 | NR | NR | Anxiety and tension improved after reserpine |
|  | Hiob et al., 1955 | Europe | Inpatient | 55 | NR | 82 | Reduction in anxiety |
|  | Pellerito, 1956 | Europe | Outpatient | ~100 | NR | NR | Reactive anxiety improvement after reserpine |
|  | Krajnakova et al., 1981 | Europe | Outpatient | 36 | 60 | 66 | - |
|  | Schwarz et al., 1973 | Europe | Inpatient | 80 | NR | NR | - |
|  | Ingrova et al., 1963 | Europe | Inpatient | 24 | Range 20-72 | 100 | - |
|  | Jeri, 1957 | S America | In/outpatient | 159 | NR | NR | - |
|  | Kirk et al., 1970 | Europe | Inpatient | 24 | NR | NR | - |
|  | Carney et al., 1969 | Europe | Inpatient | 8 | 55 | 90 | - |
|  | Bant et al., 1978 | Europe | Outpatient | 20 | 51 | 42 | - |
| **Cross-sectional** | Zhu et al., 2019 | Asia | Outpatient | 787 | 70 | 50 | - |
|  | Prisant et al., 1991 | N America | Outpatient | 111 | 56 | NR | - |
|  | Dissegna et al., 1985 | Europe | Community | 73 | 54 | 47 | NS difference in anxiety between treated/untreated |

^a^ Describes eligible depression subgroup where full sample did not meet review inclusion criteria.

^b^ Outcomes came from follow-up (follow-up period average of 13.4 months)

^c^ physician prepared questionnaire

^d^ phenobaritone, aminophyllin, ganglion-blockers, parenteral hexamethonium, combined reserpine and pentolinium

^e^ non-randomised

^f^ Hamilton Anxiety Scale

* Some improvements to (hypo)mania also reported after reserpine

*Supplementary table 2: Methodological quality and risk of bias in the included studies.*

Evaluations of the 35 included studies for potential sources of bias according to the modified ROBINS-I tool (Risk of Bias in Non-randomized Studies of Interventions; Sterne et al., 2019).

|  | **Potential sources of bias** | | | | | | | | | **Overall RoB judgement** |
| --- | --- | --- | --- | --- | --- | --- | --- | --- | --- | --- |
| **Reference** | **1** | **2** | **3** | **4** | **5** | **6** | **7** | **8** | **9** |  |
| Zhu et al., 2019 | NA | NA | + | ? | - | NA | + | + | + | High |
| Bolte et al., 1959 | NA | NA | - | ? | - | ? | - | ? | + | High |
| Bant et al., 1978 | NA | NA | - | ? | - | - | - | ? | + | High |
| Carney et al., 1969 | NA | NA | NA | NA | NA | - | - | ? | + | High |
| Davies et al., 1955 | ? | + | + | + | + | - | + | ? | + | *Moderate* |
| Finn et al., 1955 | NA | NA | ? | + | - | ? | + | ? | + | High |
| Azima et al., 1959 | ? | ? | + | + | + | + | + | ? | + | **Low** |
| Berger et al., 2005 | ? | ? | - | ? | + | + | + | - | + | High |
| Hopkinson et al., 1975 | + | ? | ? | + | + | + | ? | ? | + | **Low** |
| Kirk et al., 1970 | NA | NA | NA | NA | - | ? | - | ? | + | High |
| Prisant et al., 1991 | NA | NA | + | ? | - | NA | - | ? | + | High |
| Veselinovic et al., 2011 | + | + | + | ? | + | + | ? | ? | - | *Moderate* |
| Winhusen et al., 2007 | + | ? | + | + | + | - | + | ? | + | *Moderate* |
| Wallace, 1955 | NA | NA | - | NA | - | NA | ? | ? | + | High |
| Achor et al., 1955 | NA | NA | NA | + | + | - | + | ? | + | High |
| Sainz, 1955 | NA | NA | NA | NA | NA | NA | - | ? | + | High |
| Lemieux et al., 1956 | NA | NA | NA | ? | - | NA | - | ? | + | High |
| Lowinger, 1957 | NA | NA | + | ? | - | NA | - | ? | + | High |
| Hodgkingson, 1955 | ? | ? | ? | + | ? | ? | ? | ? | + | High |
| Platt et al., 1956 | NA | NA | NA | ? | - | ? | - | ? | + | High |
| Jal Vakil, 1949 | NA | NA | NA | NA | + | - | - | ? | + | High |
| Wachspress et al., 1956 | ? | ? | ? | + | ? | - | + | ? | + | High |
| Fife et al., 1958 | NA | NA | NA | ? | ? | - | - | ? | + | High |
| Santucci et al., 1989 | ? | ? | + | ? | + | ? | + | ? | + | High |
| Segal et al., 1959 | - | - | ? | + | + | - | + | ? | + | High |
| Drake et al., 1955 | NA | NA | NA | NA | - | NA | - | ? | + | High |
| Kirkegaard et al., 1958 | NA | NA | NA | NA | - | NA | - | ? | + | High |
| Bennett et al., 1956 | NA | NA | ? | ? | - | NA | - | ? | + | High |
| Hiob et al., 1955 | NA | NA | NA | NA | NA | NA | - | ? | + | High |
| Dissegna et al., 1985 | NA | NA | + | ? | + | NA | ? | ? | + | High |
| Pellerito, 1956 | NA | NA | ? | ? | + | NA | ? | ? | + | High |
| Krajnakova et al., 1981 | NA | NA | + | - | + | NA | ? | ? | + | High |
| Schwarz et al., 1973 | NA | NA | + | ? | + | - | ? | ? | + | High |
| Ingrova et al., 1963 | NA | NA | NA | NA | NA | NA | - | ? | + | High |
| Jeri, 1957 | NA | NA | NA | NA | NA | NA | - | ? | + | High |

1. Allocation sequence randomly generated; 2. Allocation sufficiently concealed; 3. Group comparability at baseline ensured; 4. Clinicians and participants blinded to intervention; 5. Equal treatment of groups; 6. Intention-to-treat analysis employed; 7. Knowledge of allocation adequately prevented (blinding); 8. No evidence of deviation from protocol; 9. Allegiance effect minimised.

**+ =** low risk/ **- =** high risk/ **?** = unclear risk

Where risk of bias source was not applicable to the study type, this was coded as ‘unclear’ when calculating overall bias risk. Overall risk was judged as follows:

*Low risk = <1 criteria rated high RoB and <4 unclear RoB*

*High risk = >4 criteria rated high or unclear RoB*

*Moderate risk if not meeting criteria for high or low risk of bias.*

*Supplementary table 3: Potential effect of factors that may influence reserpine effects on depression, categorised by direction and strength of reported effect*

|  | Reference | Population | Type | N | Reserpine duration | Reserpine dose | % depressed (baseline) | Primary outcome measure | ROB level | Notes |
| --- | --- | --- | --- | --- | --- | --- | --- | --- | --- | --- |
| **Poss/definitely depressogenic** | Bolte et al., 1959 | Hypertension | Nat. | Large (>100) | High (>26w) | Variable/Moderate | 0 | Non-validated | High | Depressogenic only in high doses |
|  | Drake et al., 1955 | Various psychiatric | Nat. | Small (<50) | Moderate (6-26w) | Variable/Moderate | 0 | Non-validated | High | Most depression unchanged, but 1 suicide attempt |
|  | Fife et al., 1958 | Hypertension | NRCT | Medium (50-100) | Variable | Variable/Moderate | 0 | Non-validated | High | Dose reduced due to depression |
|  | Platt et al., 1956 | Hypertension | NRCT | Medium (50-100) | Variable | Variable/Moderate | 0 | Non-validated | High |  |
|  | Veselinović et al., 2011 | Healthy | RCT | Small (<50) | Low (<6w) | Low (~<1mg) | 0 | Validated (CR) | Moderate |  |
|  | Krajnakova et al., 1981 | Hypertension | Nat. | Small (<50) | High (~>26w) | NR | 0 | Validated (PR) | High | High depression but the comparator therapies also contained rauwolfia |
|  | Lemieux et al., 1956 | Hypertension | Nat. | Large (>100) | High (>26w) | Variable/Moderate | 0 | Non-validated | High |  |
|  | Achor et al., 1955 | Hypertension | NRCT | Medium (50-100) | Moderate (6-26w) | Low (~<1mg) | 0 | Non-validated | High |  |
|  | Azima et al., 1959 | Psychosis | RCT | Small (<50) | Low (<6w) | High (>3mg) | 0 | Non-validated | Low | Mixed symptoms (mostly elation) |
|  | Jeri, 1957 | Psychosis | Nat. | Large (>100) | Low (<6w) | High (>3mg) | 0 | Non-validated | High |  |
|  | Wachspress et al., 1956 | Various psychiatric | RCT | Small (<50) | Moderate (6-26w) | High (>3mg) | 1-49% | Non-validated | High |  |
| **No effect** | Bant et al., 1978 | Hypertension | Nat. | Small (<50) | High (>26w) | Low (~<1mg) | 0 | Validated (PR) | High |  |
|  | Bennett et al., 1956 | Various psychiatric | Nat. | Medium (50-100) | Variable | Variable/Moderate | 1-49% | Non-validated | High | Reserpine facilitated psychotherapy outcome |
|  | Carney et al., 1969 | Depression | Nat. | Small (<50) | Low (<6w) | High (>3mg) | 50-100% | Validated (CR) | High | Some improvements in depression but 1 mania |
|  | Dissegna et al., 1985 | Hypertension | Cross-sec | Medium (50-100) | NR | Low (~<1mg) | 0 | Validated (PR) | High |  |
|  | Hodgkinson, 1955 | Hypertension | NRCT | Small (<50) | High (>26w) | Variable/Moderate | 0 | Non-validated | High | Minimal increases in depression observed |
|  | Jal Vakil, 1949 | Hypertension | Nat. | Medium (50-100) | Low (<6w) | NR | 0 | Non-validated | High | Minimal increases in depression observed |
|  | Lowinger, 1957 | Various psychiatric | NRCT | Medium (50-100) | Low (<6w) | Variable/Moderate | 1-49% | Non-validated | High | Rauwolfia aided psychotherapy engagement |
|  | Prisant et al., 1991 | Hypertension | Cross-sec | Large (>100) | Moderate (6-26w) | NR | 0 | Validated (PR) | High | Slightly less depression in reserpine than others |
|  | Santucci et al., 1989 | Hypertension | RCT | Small (<50) | Moderate (6-26w) | Low (~<1mg) | 0 | Non-validated | High | Less/more depression in reserpine than others |
|  | Schwarz et al., 1973 | Hypertension | Nat. | Medium (50-100) | Low (<6w) | Low (~<1mg) | NR | Validated (CR) | High | Depression improved overall but some new cases |
|  | Segal et al., 1959 | Anxiety | RCT | Small (<50) | Low (<6w) | Low (~<1mg) | 0 | Non-validated | High | Equivalent to placebo |
|  | Wallace, 1955 | Hypertension | Nat. | Small (<50) | High (>26w) | NR | 0 | Non-validated | High | Reserpine monotherapy equivalent to others |
|  | Zhu et al., 2019 | Hypertension | Cross-sec | Large (>100) | High (>26w) | Low (~<1mg) | 0 | Validated (PR) | High |  |
| **Potentially antidepressant** | Berger et al., 2005 | Cocaine dependence | RCT | Small (<50) | Moderate (6-26w) | Low (~<1mg) | 0 | Validated (CR) | High | Participants received CBT. |
|  | Davies et al., 1955 | Anxiety and depression | RCT | Small (<50) | Low (<6w) | Low (~<1mg) | 50-100% | Non-validated | Moderate |  |
|  | Finn et al., 1955 | Psychosis | NRCT | Small (<50) | Moderate (6-26w) | Variable/Moderate | 0 | Validated (CR) | High |  |
|  | Hiob et al., 1955 | Various psychiatric^a^ | Nat. | Medium (50-100) | Low (<6w) | High (>3mg) | 1-49% | Non-validated | High |  |
|  | Hopkinson et al., 1975 | Depression | RCT | Small (<50) | Low (<6w) | Variable/Moderate | 50-100% | Validated (CR) | Low |  |
|  | Ingrova et al., 1963 | Various psychiatric | Nat. | Small (<50) | Low (<6w) | High (>3mg) | 1-49% | Non-validated | High |  |
|  | Kirk et al., 1970 | Depression^a^ | Nat. | Small (<50) | Low (<6w) | High (>3mg) | 50-100% | Non-validated | High |  |
|  | Kirkegaard et al., 1958 | Various psychiatric^a^ | Nat. | Large (>100) | Variable | Variable/Moderate | 1-49% | Non-validated | High |  |
|  | Pellerito, 1956 | Various psychiatric | Nat. | Medium (50-100) | Moderate (6-26w) | Variable/Moderate | 0 | Non-validated | High |  |
|  | Sainz, 1955 | Depression^a^ | Nat. | Small (<50) | Moderate (6-26w) | High (>3mg) | 50-100% | Non-validated | High |  |
|  | Winhusen et al., 2007 | Cocaine dependence | RCT | Small (<50) | Moderate (6-26w) | Low (<1mg) | 0 | Validated (CR) | Moderate | Patients received CBT |

^a^ Although not ostensibly depressed at baseline, anxiety patients with prominent depression had a worse response to reserpine

Results are categorised by strength and direction of reserpine-depression finding, from those reporting reserpine as definitively depressogenic (6 studies), potentially depressogenic (8 studies), no influence (11 studies) to a potential antidepressant effect (10 studies).

Green cells indicate factors that may minimise risk (non-psychiatric population, controlled trial, large sample, short duration, low dose, validated measure of depression, low ROB). Red cells indicate studies at risk of finding an effect due to participant risk factors (e.g. pre-existing psychiatric illness), methodology (uncontrolled, judgement-based depression measure, high ROB) or treatment factors (long duration or high dose). Yellow represents a midway for each category and grey indicates a lack of information required to determine risk level.
